# Recent advances of eosinophils and its correlated diseases

**DOI:** 10.3389/fpubh.2022.954721

**Published:** 2022-07-25

**Authors:** Zhang Tao, Hua Zhu, Jiateng Zhang, Zhiming Huang, Ze Xiang, Tu Hong

**Affiliations:** ^1^Department of Pulmonary Diseases, Yancheng Traditional Chinese Medicine Hospital, Yancheng, China; ^2^Department of Gastroenterology, Yancheng Third People's Hospital, Yancheng, China; ^3^School of Medicine, Affiliated Yancheng Hospital, Southeast University, Yancheng, China; ^4^Zhejiang University School of Medicine, Hangzhou, China; ^5^Chu Kochen Honors College of Zhejiang University, Hangzhou, China

**Keywords:** eosinophils, cellular functions, molecular mechanisms, disease pathology, therapeutic drugs

## Abstract

Eosinophils are differentiated by bone marrow multipotent progenitor cells and are further released into peripheral blood after maturation. Human eosinophils can exhibit unique multi-leaf nuclear morphology, which are filled with cytoplasmic granules that contain cytotoxicity and immune regulatory proteins. In recent years, many studies focused on the origin, differentiation and development process of eosinophils. It has been discovered that the eosinophils have the regulatory functions of innate and adaptive immunity, and can also function in several diseases, including asthma, chronic obstructive pulmonary diseases, acute respiratory distress syndrome, malignant tumors and so on. Hence, the role and effects of eosinophils in various diseases are emphasized. In this review, we comprehensively summarized the development and differentiation process of eosinophils, the research progress of their related cytokines, diseases and current clinical treatment options, and discussed the potential drug target, aiming to provide a theoretical and practical basis for the clinical prevention and treatment of eosinophil-related diseases, especially respiratory diseases. To conclude, the guiding significance of future disease treatment is proposed based on the recent updated understandings into the cell functions of eosinophils.

## Introduction

Eosinophils are differentiated by bone marrow multipotent progenitor cells and are further released into peripheral blood after maturation under the actions of interleukin-5 (IL-5) and interleukin-33 (IL-33). After a short stay in peripheral blood, eosinophils migrate to the lungs, thymus and gastrointestinal tract (1, 2).

During the occurrence and development of related diseases, eosinophils are recruited to the disease lesions and exert their cellular functions under the influence of the local microenvironment. It has been reported that eosinophils play an effective role in allergic diseases and anti-parasitic infections (3). Recently, studies have explored inflammatory cytokines and biomarkers in several diseases (4, 5). The further discovery of different eosinophil subtypes and related cytokines and media has enriched the cognition of their cellular functions, including anti-tumor effects, regulation of hematopoietic stem cell homeostasis (6, 7).

In this review, we summarized the development and differentiation process of eosinophils, the research progress of their related cytokines, diseases and current clinical treatment options, and discussed the potential drug target, aiming to provide a theoretical and practical basis for the clinical prevention and treatment of eosinophil-related diseases, especially respiratory diseases.

## Origin and differentiation of eosinophils

**Figure 1:**
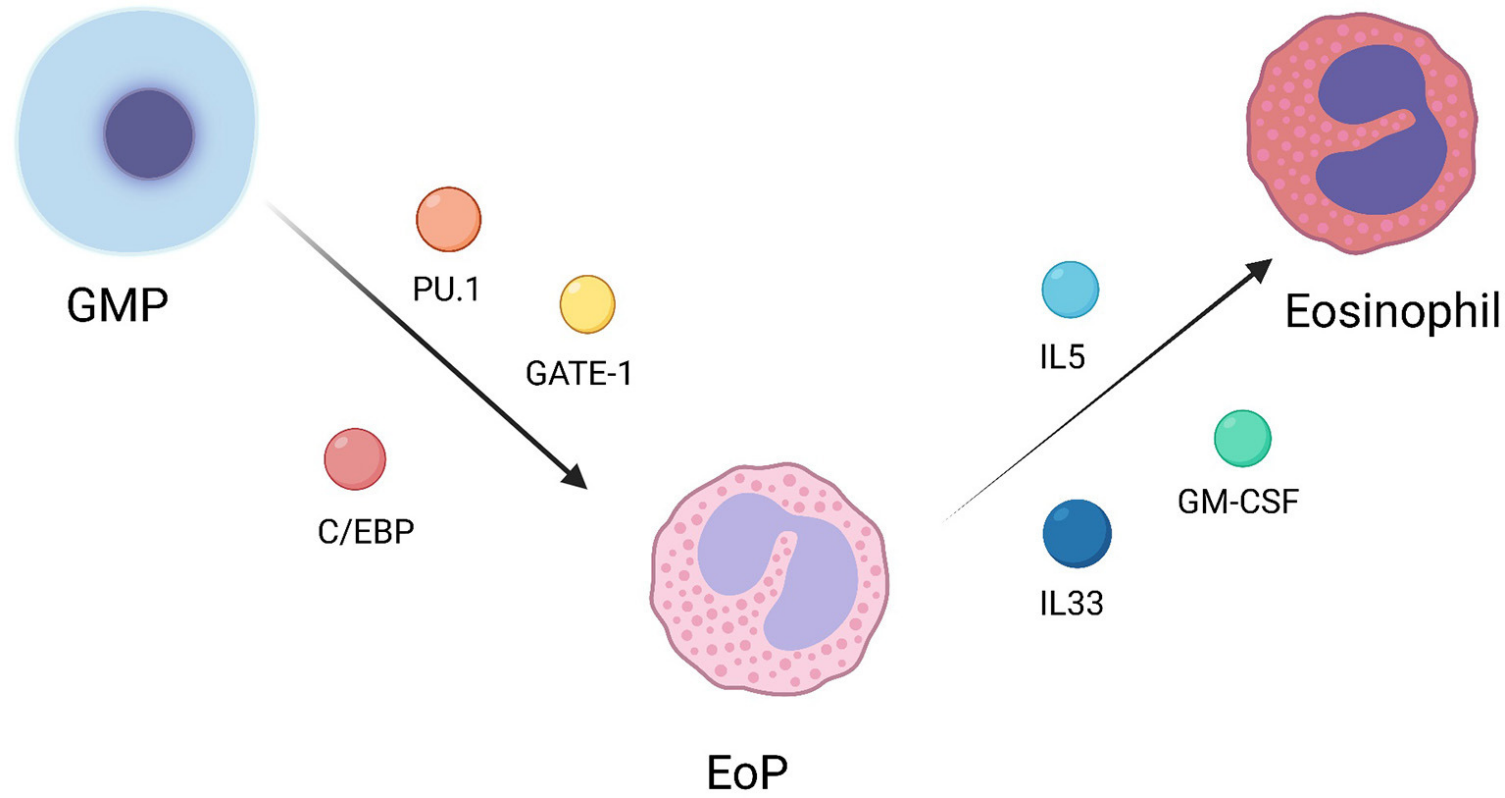
Differentiation of mature eosinophils.

Eosinophils, along with neutrophils and basophils, are major members of granulocytes. Granulocyte/monocyte progenitor (GMP) is their common progenitor. GMP could differentiate into eosinophile lineage-committed progenitor (EoP) under the regulation of transcription factors, including PU.1, C/EBP, and GATA-1. Later, EoP continues to differentiate into mature eosinophils under the regulation of GM-CSF, IL-5 and IL-33 (Figure 1) (8). In addition, it has been reported that some regulatory factors could effectively regulate eosinophil differentiation. For instance, eosinophil differentiation can be inhibited by rapamycin through a pathway independent of the IL-5 signaling pathway in mice (9). Xia et al. demonstrated that protein tyrosine phosphatase SHP2 could regulate IL-5 levels through Erk signaling pathway to regulate the differentiation of eosinophils (10). Exogenous interleukin-17A (IL-17A) could also inhibit the differentiation of eosinophils (11).

## Eosinophil-related molecules

### Particulate proteins from eosinophil-derived granules

Eosinophil-derived granule proteins (EDGPS) mainly consist of main basic protein (MBP), eosinophil cation protein (ECP), eosinophil-derived neurotoxin (EDN), and eosinophilic peroxidase (EPO). MBP is located at the center of the granules and exists in the form of two homologous proteins, MBP-1 and MBP-2. MBP is small in size and consists of a single chain of 117 amino acid residues, which is highly alkaline that is toxic to both parasites and bacteria (12). ECP and EDN are initially found to be acidophilic granulocyte-related RNA enzymes that are associated with neurotoxicity. They can exhibit RNA hydrolyze capabilities, and their homologous gene sequences only exist in primate genomes (13). ECP is a single-stranded cation protein that is homologous to lychee RNA, which exhibit toxicity to worms and can bind to bacterial cell wall components such as lipid polysaccharides (14). In addition, ECP can also affect the proliferation of T lymphocytes and B lymphocytes, thereby promoting the degranulation of mast cells, and regulate the classical activation pathway of complements (15). EDN is a single-stranded peptide with antiviral properties and the ability to degrade single-stranded RNA, which can also serve as a biomarker for the activation and degranulation of eosinophils in patients with asthma (16, 17). EPO is a hemoglobin-containing halogen peroxidase that catalyzes the reactions of halides present in plasma and pseudohalides, which promote asthma-related characteristic phenotypes through post-translational modification of proteins in the airway of asthma using carbonylation (6, 18).

### Cytokines associated with eosinophils

Eosinophils can synthesize, store and secrete various cytokines. In eosinophils, many cytokines, chemokines and growth factors are stored preformed in particles, ready for immediate release, unlike other granulocytes. Specific particles are rich in cytokines, including IL-2, IL-3, IL-4, IL-5, IL-6, IL-13, IL-33, interferon-γ (IFN-γ), GM-CSF, and tumor necrosis factor-α (TNF-α). Convergence factors include RANTES, Eotaxin, and MIP-1 alpha, while growth factors include stem cell factors and transforming growth factors (TGF) (Table 1).

**Table 1:** Eosinophils-related cytokines and their roles.

**Cytokines**	**Related pathway and molecules**	**Roles to eosinophils**
IL-3	-	Functioning in allergic inflammation by activating eosinophils and basophils.
	CD32, CD13, CD48 and so on	Promoting the expression of eosinophil proteins.
	-	Correlated with the levels of eosinophil granule proteins.
IL-5	Pim-1, c-fos, c-jun and NF-κb	Regulating the survival, immune response and proliferation differentiation.
	T helper 2 and CD34+	Functioning in Th2 immune response and CD34+ ancestral cells differentiation.
IL-33	ST2	Inducing a type 2 immune response.
	ST2/IL-33 axis	Increasing the number of eosinophiles.
CCL11/Eotaxin	-	Inducing the fetching of eosinophils in allergic reactions.
	-	Reducing allergic inflammatory reactions in the intestines, skin and airways.
	Ca^2+^	Activating intracellular Ca^2+^ activity, granules and respiratory bursts.
IL-13	-	Activating the matrix metalloproteinase to prevent excessive allergic inflammation.
	B cells	Inducing antibody type conversion in B cells to produce IgE.

#### IL-3

IL-3 can promote the production of not only eosinophils, basophils and mast cells, but also granulocytes, monocytes, and macrophages. For the ability to drive the entire myelopoiesis spectrum, IL-3 was firstly known as multi-CSF (19). T cells and mast cells are the main cellular sources of IL-3 (20). IL-3 takes a great important part in allergic inflammation by activating eosinophils and basophils (21). Eosinophils are strongly correlated with IL-3, it is known that IL-3 and GM-CSF play essential roles in the early stage of eosinophil differentiation, and IL-5 plays a role in the end stage of eosinophil maturation (22, 23). Compared to IL-5 and GM-CSF, several studies have shown that IL-3 is more able to promote the expression of eosinophil proteins like CD32, CD13, CD48 and so on (24). In addition, a research has demonstrated that the amounts of sputum IL-3 are strongly correlated with levels of eosinophil granule proteins, and decreased lung function (25).

#### IL-5

IL-5 is an important cytokine and has exhibit many physiological functions, including prolongation of survival, induction, activation, and degranulation of eosinophils. Eosinophils could express IL-5Rα at high level on their surface and release a large amount of IL-5 (26). When IL-5 is engaged with IL-5R, a range of proteins are induced phosphorylation, which further activating Pim-1, c-fos, c-jun and NF-κb that could regulate the survival, immune response, and proliferation differentiation of eosinophils (27–29). However, it has been shown that a systemic increase in IL-5 does not necessarily lead to pathological conditions that are mediated by eosinophils (30). Besides, IL-5 plays an important role in the development of the Th2 immune response and is essential for the differentiation of CD34+ ancestral cells into eosinophils (31, 32).

#### IL-33

IL-33 belongs to the IL-1 cytokine family and is a ligand of the transmembrane protein ST2 encoded by the IL-1rl1 gene (33). In a stable state, IL-33 is located in nuclei and is associated with chromoplast by chromoplast binding matrix sequence, which promotes cell stability by serving as a transcription inhibitor (34). Since IL-33 does not contain a signal sequence, the secretion manner of IL-33 is different from conventional cytokines (35). After mechanical damage, necrosis cell death, and the activation of cells through the ATP signaling pathway without cell death, IL-33 would be released into extracellular space (36, 37). By activating immune cells that express ST2 in the mucosa organs throughout the body, IL-33 could induce a type 2 immune response, which promotes the growth of blood and tissue eosinophils by IL-5-mediated pathway (33, 38–41). Johnston et al. revealed that the ST2/IL33 axis is the best way for proliferation of eosinophils, and this can be achieved by raising the surface IL-5Rα of eosinophils (41). IL-33 expression is closely related to the increase of peripheral hemophilic granulocytes. Smith et al. demonstrated that IL-33 gene sequence led to a decrease of IL-33 protein levels. In addition, a decrease in the number of peripheral blood eosinophils in mice was also observed (42).

#### CCL11/Eotaxin

CCL11/Eotaxin is an important acidophil-specific chemokine, which is involved in the chemotaxis of eosinophils to tissue, and could serve as an efficient activator inducing the fetching of eosinophils in allergic reactions (43). The expression of CCL11/Eotaxin in eosinophils is associated with intracellular particles (44). knocking out the CCL11/Eotaxin gene could significantly reduce the accumulation of eosinophils in tissues, thus reducing allergic inflammatory reactions in the intestines, skin, and airways (45). Similar to CCL5/RANTES, CCL11/Eotaxin activates intracellular Ca^2+^ activity, granules, and respiratory bursts in eosinophils, indicating that it processed in a self-secreting manner (46, 47).

#### IL-13

IL-13 is another important inflammatory factor released by eosinophils, which is stored in particle crystallization as a pre-formed medium. IL-13 has an impact on the development of asthma airway disease and pulmonary fibrosis, and it could also activate the matrix metalloproteinase in the airways to prevent excessive allergic inflammation (48). IL-13 can also induce antibody type conversion in B cells to produce IgE, which plays an important role in allergic inflammation (49, 50). In addition, parasites such as worms are also dependent on IL-13 for intestinal discharge from mice (51).

## Eosinophil-related diseases

### Asthma

Asthma is a common chronic airway inflammatory disease, in which allergen-induced asthma occupies the majority. The pathological characteristics of asthma are the accumulation and activation of eosinophils in the airways. The clinical characteristics mainly include chronic inflammation, reversible airflow restriction, high mucus secretion, high bronchial reactive accompanied by cough, sputum, wheezing, and other clinical symptoms (52). It was found that the levels of eosinophils in peripheral blood in asthma patients are closely related to the severity of asthma (53, 54).

Elevated levels of eosinophils in peripheral blood commonly represent severe asthma, also known as T helper 2 (Th2) asthma (55). Since the granulocyte proteins of eosinophils could coordinate the immune response to worms in the Th2 cytokine cascade response, eosinophils are primarily related to parasitic infection (56). The cascade starts with the reaction of IgE and antigens. Although antigens could pose a threat to the host in worm infection, the targets of IgE in patients with asthma are relatively harmless, such as tree pollen and animal fur. However, IgE can activate hypertrophic cells, macrophages, and basophils, which in turn leads to the production of histamines and other inflammatory cytokines secretion. The airway inflammatory microenvironment of asthma has a strong chemoattraction to mature CD4+T cells and eosinophils, which leads to severe type Th2 asthma and is accompanied with high levels of eosinophils in blood and sputum (57). In recent years, it was also found that patients with classic asthma, cough-mutant asthma, and chest tightness variant asthma usually have the common clinical feature of eosinophilic airway inflammation. The recruitment of mature eosinophils from peripheral circulation may be the main mechanism for the growth of pulmonary eosinophils (58). Additionally, circulating progenitor cells were also proved to accumulate in the inflammatory site and differentiate into mature immune cells to promote tissue inflammation (59–61).

### Acute lung injury and acute respiratory distress syndrome

Acute lung injury (ALI) and acute respiratory distress syndrome (ARDS) are characterized by increased pulmonary vascular permeability, severe inflammation, secondary pulmonary edema, and refraction of hypoxia (62, 63). During the progression of ALI and ARDS, the accumulation of inflammatory cells and cytokines leads to the destruction of the capillary endothelium and alveolar epithelial barrier, which subsequently promote the development of pulmonary edema and hypoxia (64). Willetts et al. concluded that patients with a good prognosis of ALI have an increased number of eosinophils in the lung compared to patients with a poor prognosis (65). Zhu et al. revealed that early-induced and period-stable eosinophils of ALI are located in the lung parenchyma, and these eosinophils recruited from peripheral blood are derived from the bone marrow. Notably, the study found that CD101 can serve as a marker for distinguishing between different eosinophil subtypes in lipopolysaccharide (LPS)-induced ALI animal models. And that under normal circumstances, CD101-negative eosinophils are predominant. In addition, CD101-negative and CD101-positive eosinophils exert different cellular effects on the inflammatory response. CD101-negative eosinophils could reduce LPS-induced early white blood cell aggregation and cytokine production, while CD101-positive eosinophils may increase LPS-induced early white blood cell aggregation and cytokine production (66).

### Eosinophil pneumonia

Eosinophil pneumonia can be divided into acute and chronic eosinophil pneumonia. Acute eosinophilic pneumonia (AEP) is commonly considered to be secondary to allergic reactions to irritants and drugs, which is rarely thought to be caused by parasitic infection (67, 68). Patients with AEP can recover on their own without external intervention, and thus eliminating adverse environmental exposure is the main treatment (68). The distinctive feature of chronic eosinophilic pneumonia (CEP) is eosinophils infiltration into the alveolar cavity and pulmonary interstitium.

### Tumor occurrence and metastasis

The relationship between eosinophils and tumors can be traced back to 1893 (69). An increase in tumor-related eosinophils was found in tumor tissues. Several studies showed an increase in tumor-associated tissue eosinophilia (TATE), mainly in colon tumors, esophageal squamous cell carcinoma, nasopharyngeal cancer, penile cancer, laryngeal cancer, lung adenocarcinoma, bladder cancer, and prostate cancer (70–74). Eosinophils are associated with necrotic regions, and there is evidence that eosinophils have cytotoxic effects on tumor cells both *in vivo* and *in vitro*. Through the observation of granule protein near the tumor, Caruso et al. demonstrated that eosinophils produce anti-tumor cytotoxic reactions through degranulation, but the tumor-killing mechanism of eosinophils remains unclear. A mouse tumor model with increased peripheral eosinophils was accompanied with inhibition of tumor development (75). In contrast, mice lacking eosinophils showed increased tumor progression, which was associated with a decrease in the number of tumor eosinophils. In humans, an increase in the number of eosinophils is often observed after immunotherapy such as IL-2, IL-4, GM-CSF, and tumor vaccine (76–80).

## Therapeutic drugs targeting eosinophils

### Glucocorticoids

Glucocorticoids (GC) are important therapeutic drugs for acidophil-related diseases such as allergies, asthma, acidophil gastrointestinal diseases (81, 82). Glucocorticoid receptors (GR) come from the NR3C1-nuclear receptor sub-family 3, and the GC diffuses through the cell membrane, enters the cell, and binds to its receptor GR to induce the activation of the GR signaling pathway. Once activated, GR is transferred to the nucleus and interacts with transcription factors to inhibit the expression of inflammatory genes and enhance the expression of anti-inflammatory genes (83, 84). GR mainly exists in cells in the airways in the form of GRα. The existence of GR explains the presence of GC in the airways of most asthma patients, as well as the significant effects of GC on inflammatory cells and therapeutic effects on asthma (85, 86). Glucocorticoids could promote the clearance of eosinophils by directly inducing apoptosis and suppressing survival signals of eosinophils *via* cytokines such as IL-3, IL-5, and GM-CSF (87, 88). Of note, Shen et al. demonstrated that corticosteroids not only play an anti-inflammatory role by regulating the release of IL-5 and pulmonary eosinophils but also inhibit bone marrow eosinophil production (89). IL-5 in eosinophils was also proved to protect against glucocorticoid-induced apoptosis (90). Additionally, Wu et al. concluded that GC can also act in synergy with iron death inducers to induce the death of eosinophils in airway inflammation (91).

### Eosinophil regulation mediated by cytokines

#### Monoclonal antibodies targeting IL-5 and IL-5R

IL-5 is considered to be a key regulator of disease-related eosinophils and has an impact on the developmental stages of multiple eosinophil lineages (92). The specific receptor of IL-5, IL-5R is expressed at all developmental stages of the eosinophil lineage. Therefore, IL-5 affects all stages of eosinophil maturation, from the proliferation and differentiation of EoP to survival and activation of mature eosinophils (93, 94).

Several studies showed that treatment targeting IL-5 could significantly reduce levels of mature eosinophils, but have little effect on the levels of EoP (95, 96). To date, two anti-IL5 drugs, meperizumab and relizumab have been approved by FDA. The two monoclonal antibodies are recombinant humanized monoclonal antibodies that could inhibit the binding of IL-5 and IL-5Rα. Biologics targeting IL-5 is designed to reduce the survival of acidophils in tissues. Although the two monoclonal antibodies are effective in reducing peripheral eosinophils, they are less effective in eliminating tissue eosinophils (97, 98). Because of the decrease in eosinophils, mepolizumab and reslizumab improve the symptoms of severe asthma, they provide significant and clinically relevant improvements in exacerbation rate and oral corticosteroid (OCS) reduction, thus greatly improve the clinical treatment of asthma (99).

For IL-5Rα, FDA has approved a biologic agent targeting IL-5 receptors called benazizumab. Benazizumab is also a humanized monoclonal antibody that could reduce eosinophils through a cell-mediated cytotoxic pathway (100). Therefore, benazizumab can directly kill eosinophils, which was shown to significantly inhibit the increase of eosinophils in tissue and thereby improve clinical manifestations (101, 102).

Intriguingly,Both meperizumab and benralizumab can significantly reduce the counts of peripheral eosinophils in eosinophilic asthma, a recent study has demonstrated that compared to meperizumab, benralizumab is able to decrease peripheral eosinophil counts in more patients (103).

#### IL-33/ST2 axis regulator

IL-33 plays an important role in airway diseases (37, 39). The levels of IL-33 are closely associated with the severity of asthma (104, 105). The regulation of the IL-33/ST2 axis is a representative treatment strategy for immune disorders associated with cytokine signaling disorders. In the past two decades, the treatment strategies blocking the IL-33/ST2 axis have been widely used in animal model diseases. Blocking the IL33/ST2 axis is protective in allergic diseases, especially in the respiratory system. There are three main therapeutic strategies for directly blocking the binding of IL-33 to ST2, including IL-33 neutralizing antibodies, soluble decoy receptors, and anti-ST2 receptor antibodies.

Several neutralizing antibodies against IL-33 have been developed, which have been used in clinical trials for the treatment of allergic diseases. Soluble receptor antagonists were also developed to bind free IL-33. So far, at least two IL-33 bait receptors have been developed, including a form of soluble ST2 (sST2) and the fusion protein IL-33 Trap, formed by sST2 and the secondary protein IL-1RAcP. Anti-ST2 could be used to treat chronic obstructive pneumonia (106). In addition to blocking the IL-33/ST2 signaling pathway, activating the IL-33 signaling pathway by recombinant IL-33 recombinant also has certain therapeutic effects in some disease models (107).

### Current drug development directions and potential targets

For eosinophil-related diseases, targeted therapies have gradually become a hot topic. Many drugs are in the process of research and put into clinical trials.

A recent study showed that exogenous IL-17A can significantly reduce ovalbumin-induced allergic inflammation. It was found that the down-regulated expression of CC chemokine receptors 3 (CCR3), GATA binding proteins 1 (GATA-1), and GATA binding proteins 2 (GATA-2) could inhibit the differentiation of eosinophils both *in vivo* and *in vitro*, suggesting that exogenous IL-17 is most likely to prevent allergic airway inflammation by inhibiting eosinophil differentiation in the bone marrow (11). This study highlights the importance of targeted inhibition of eosinophil differentiation in allergic inflammation, which may be a new therapeutic target for asthma.

It was also found that the elevated levels of Bcl-2 protein are responsible for the persistence of eosinophils and neutrophils during allergic airway inflammation Furthermore, the Bcl-2 inhibitor ABT-737 and ABT-199 may inhibit allergic airway inflammation by promoting the death of inflammatory cells, especially in asthma dominated by neutrophils and insensitive to corticosteroids (108). Notably, nano Bcl-2 inhibitor, Nf-ABT-199 could deliver ABT-199 specifically to mitochondria of bronchitis cells, which was proved to significantly reduce airway inflammation and inhibit inflammatory cell infiltration and mucus excessive secretion by effectively inducing eosinophil apoptosis. In addition, Nf-ABT-199 had no significant effect on cell vitality, airway epithelial barrier, and liver function, indicating non-toxicity and good biocompatibility (109).

Iron death inducers were explored to induce iron death of eosinophils in mice, thereby alleviating eosinophils airway inflammation. Interestingly, iron death inducers could act in synergy with dexamethasone to induce the death of eosinophils (91). The synergistic effect of iron death inducers and glucocorticoids has advantages in the treatment of allergic diseases, showing that iron death inducer may be a promising treatment strategy for acidophilic airway inflammation.

Moreover, CCL6 expression was significantly increased in asthma patients. The OVA model of asthma was constructed using the CCL6 knockout mouse model, which significantly reduced airway inflammation in mice. The CCL6-CCR1 axis was involved in the process of differentiation from HSC to eosinophils, and the use of specific CCR1 antagonist BX471 can significantly inhibit eosinophil differentiation in both *in vitro* and *in vivo* experiments (110). In addition, there exists evidence that mCCL6 could activate CCR1 downstream of the Gαi protein and related phosphorylated signaling proteins, thereby promoting HSC differentiation, which provides sufficient evidence for the involvement of the CCL6-CCR1 axis. Recently, several CCR1 antagonists have been developed in the context of inflammatory diseases, which have shown potential therapeutic effects in clinical trials.

## Conclusions and prospects

Nowadays, research on the origin, differentiation and development process of eosinophils has been relatively mature. The role and effects of eosinophils in various diseases have been explored. Eosinophils play an important pathophysiological role in the body due to its unique degranulation and related secreted cytokines, and when it functions, it can cause the body to produce corresponding symptoms. In recent years, the research on different cytokines in diseases has made great progress (111, 112).

The diseases most associated with eosinophils include asthma, acute lung injury/acute respiratory distress syndrome, eosinophil pneumonia, parasitic infection and esophagitis. Due to the universality of respiratory diseases, more attention is paid to the exploration of respiratory diseases and the development of corresponding drugs in clinical practice. In terms of drugs for controlling eosinophils, they could be divided into traditional glucocorticoids and cytokine regulators. Glucocorticoids focus on inducing eosinophil apoptosis, while cytokine regulators focus on blocking and interference of classical cytokines and related pathways. Most future drug development directions and targets related to eosinophils are derived from the abnormally expressed cytokines and proteins in eosinophil-related diseases, and also from the study of cell death mechanisms such as iron death.

Generally speaking, in order to develop more eosinophil-related drugs in the future, the first step is to deepen the research on the origin, development and differentiation of eosinophils. Combined with the development of single cell sequencing technology, the upstream progenitor cells of eosinophils can be grouped to further explore the origin of eosinophils. Secondly, we should study the cytokines related to eosinophils more extensively, and more research on cytokines can better provide a wide range of targets for eosinophils. Furthermore, it is also a key point to promote the research of eosinophil-related drugs by further accelerating clinical trials and allowing more drugs to show their curative effects and give feedback as soon as possible, so as to provide reference value and development direction for more similar drugs.

## Author contributions

ZT and HZ had the idea for the article. JZ and ZH performed the literature search and data analysis. ZX and TH drafted and critically revised the work. All authors contributed to the article and approved the submitted version.

## Conflict of interest

The authors declare that the research was conducted in the absence of any commercial or financial relationships that could be construed as a potential conflict of interest.

## Publisher's note

All claims expressed in this article are solely those of the authors and do not necessarily represent those of their affiliated organizations, or those of the publisher, the editors and the reviewers. Any product that may be evaluated in this article, or claim that may be made by its manufacturer, is not guaranteed or endorsed by the publisher.
